# Cerebrospinal Fluid Findings among Patients with Anaplasmosis and Central Nervous Involvement, Minnesota and Wisconsin, USA

**DOI:** 10.3201/eid3206.260240

**Published:** 2026-06

**Authors:** Igor Dumic, Charles W. Nordstrom, Marie Schulz, Sagar B. Dugani, John Fox, Ronin Joshua Cosiquien, Tatjana Gavrancic, Margaret Paulson, Cristian Madrid, Wendelyn Bosch

**Affiliations:** Mayo Clinic College of Medicine and Science, Rochester, Minnesota, USA (I. Dumic, C.W. Nordstrom, T. Gavrancic, M. Paulson, C. Madrid, W. Bosch); Mayo Clinic Health System, Eau Claire, Wisconsin, USA (I. Dumic, C.W. Nordstrom, M. Paulson, C. Madrid); Medical College of Wisconsin–Central Wisconsin, Wausau, Wisconsin, USA (M. Schulz, J. Fox); Mayo Clinic, Rochester (S.B. Dugani); University of Minnesota Twin Cities, Minneapolis, Minnesota, USA (R.J. Cosiquien); Mayo Clinic, Jacksonville, Florida, USA (T. Gavrancic, W. Bosch)

**Keywords:** Anaplasmosis, Anaplasma phagocytophilum, vector-borne infections, bacteria, zoonoses, cerebrospinal fluid, tickborne disease, meningitis/encephalitis, Minnesota, Wisconsin, United States

## Abstract

Anaplasmosis, an emerging tickborne zoonosis, infrequently involves the central nervous system, and cerebrospinal fluid (CSF) profiles of anaplasmosis remain poorly characterized. We conducted a multisite retrospective study of patients hospitalized with anaplasmosis during November 1, 2014–November 29, 2024, in Minnesota and Wisconsin, USA, a hyperendemic region. Included patients had anaplasmosis confirmed by PCR on blood samples, exhibited neurologic symptoms, and had lumbar puncture procedures. Ten hospitalized patients met inclusion criteria, 6 with meningitis, 3 with meningoencephalitis, and 1 with encephalitis. CSF findings were within reference ranges for 5 patients; 4 patients demonstrated mild lymphocytic pleocytosis, but glucose and protein levels were within reference ranges. One patient underwent a traumatic lumbar puncture resulting in neutrophilic pleocytosis. CSF abnormalities did not correlate with neurologic severity, suggesting a cytokine-mediated process rather than direct central nervous system infection. All patients rapidly improved with doxycycline, highlighting the need for early recognition and empiric therapy for anaplasmosis.

Anaplasmosis is an emerging tickborne zoonosis that has substantially increased in incidence during the past 2 decades. That rise is attributed to improved diagnostic testing, heightened clinician awareness, and climate-related factors, mirroring trends observed with other vectorborne diseases that depend on vector survival for transmission (*1*–*6*). Anaplasmosis exhibits a wide spectrum of clinical manifestations, and common symptoms include fever, chills, rigors, headache, malaise, and myalgia. Laboratory abnormalities frequently include leukopenia, thrombocytopenia, and elevated transaminases (*1**–**6*). Patients with altered mental status, immunosuppression, advanced age, or multiple underlying conditions are at increased risk for hospitalization (*1*,*5*).

Although rare, neurologic manifestations of anaplasmosis can include encephalitis, meningitis, meningoencephalitis, focal paralysis, and stroke (*6*–*10*). Case reports describing patients with central nervous system (CNS) symptoms document variable cerebrospinal fluid (CSF) findings. In some instances, CSF profiles are within reference ranges, whereas other cases demonstrate variable pleocytosis, proteinorachia, or glycorrhachia (*8**–**16*).

Most data describing CSF profiles in CNS anaplasmosis have been limited to individual case reports (*8**–**16*), and retrospective studies or case series have not systematically evaluated CSF findings in patients primarily exhibiting CNS manifestations. Thus, we sought to characterize the CSF findings of patients hospitalized with anaplasmosis who exhibited predominantly CNS symptoms and underwent lumbar puncture (LP) and CSF analysis in an anaplasmosis hyperendemic region in Minnesota and Wisconsin, USA.

## Methods

We conducted a retrospective chart review of adult patients with diagnosed *Anaplasma phagocytophilum* infection (International Classification of Diseases, 9th Revision, codes A77.49 or A79.82) who were admitted to Mayo Clinic Rochester or Mayo Clinic Health System sites in Minnesota and Wisconsin during November 1, 2014–November 29, 2024. The study was approved by the Mayo Clinic Institutional Review Board (approval no. 18-007901).

We manually reviewed a total of 101 patient charts. We included patients >18 years of age if they had a positive *A. phagocytophilum* PCR performed on EDTA-anticoagulated whole blood and predominantly had CNS symptoms when care was sought that prompted LP and CSF analysis.

We performed molecular detection by using the automated MagNA Pure 96 system (Roche Diagnostics) for DNA extraction, followed by amplification of a conserved region of the *gro*EL heat shock protein operon gene. We identified organisms via melting curve analysis by using the LightCycler 480 Instrument II (Roche Diagnostics). That assay uses fluorescence resonance energy transfer probes to detect and differentiate *A. phagocytophilum*, *Ehrlichia chaffeensis*, *E. muris eauclairensis*, and *E. ewingii/canis* (*1*).

All patients underwent standard CSF testing including a meningitis/encephalitis panel per institutional protocol. We defined CNS symptoms as severe headache, confusion or altered mental status, central focal neurologic deficits, or signs of meningitis (including neck stiffness, photophobia, or positive meningeal signs).

## Results

Of 101 hospitalized patients with anaplasmosis diagnoses, 10 (10%) met inclusion criteria. Among those, 2 patients were immunocompromised and 4 required intensive care unit (ICU) admission. Six patients had clinical features consistent with meningitis, 3 with meningoencephalitis, and 1 with encephalitis (Table 1). All 10 patients underwent extensive infectious evaluation for alternative bacterial, viral, endemic fungal, and tickborne etiologies (Appendix Table), including testing for other *Ixodes scapularis* tick–transmitted pathogens. All of those results were negative, thereby excluding co-infection. 

**Table 1:** Characteristics and cerebrospinal fluid findings among patients with anaplasmosis and central nervous system involvement, Minnesota and Wisconsin, USA*

Patient no.	Age, y/sex	Immune status	Neurologic manifestation	Sepsis	ICU admission	Cerebrospinal fluid results				
						Color	Leukocyte count†	Leukocyte type (%)	Protein, mg/dL‡	Glucose, mg/dL§
1	75/M	IC	Left hemiparesis, meningitis	N	N	Clear	5	Lymphocyte (40)	48	56
2	65/F	IC	Meningitis	Y	Y	Clear	1	Lymphocyte (78)	33	81
3	74/F	IC	Meningitis, akathisia	N	N	Clear	1	Lymphocyte (83)	33	67
4	57/M	IS	Meningitis	N	N	Clear	0	NA	31	61
5	57/M	IS	Meningitis	Y	Y	Clear	50	Lymphocyte (78)	40	58
6	67/F	IC	Meningitis	N	N	Clear	1	Lymphocyte (72)	34	66
7	60/M	IC	Meningoencephalitis	N	N	Clear	15	Lymphocyte (43); neutrophils (30)	27	53
8	60/F	IC	Meningoencephalitis	Y	Y	Bloody	198	Neutrophils (71)	18	60
9	70/F	IC	Meningoencephalitis	N	N	Clear	1	Lymphocyte (72)	33	80
10	70/M	IC	Encephalitis	Y	Y	Clear	10	Lymphocyte (50)	189	43

*IC, immunocompetent; ICU, intensive care unit; IS, immunosuppressed; NA, not applicable. †Reference range 0. ‡Reference range <50 mg/dL. §Reference range 36–101 mg/dL.

In all cases, LP was performed before initiation of doxycycline therapy. Five (50%) patient samples demonstrated pleocytosis, and the other 5 had CSF cell counts within reference ranges. Of the 5 patients with pleocytosis, 4 had mild lymphocytic pleocytosis (5–50 cells/μL [reference range 0–5 cells/μL]) and 1 had neutrophilic pleocytosis attributable to traumatic LP. CSF protein and glucose levels were within reference ranges in all patients except for patient 8, who had a traumatic LP.

### Patient 1

A 75-year-old immunocompetent man was admitted with fever, headache, photophobia, chills, left-sided hemiparesis, and urinary retention. Laboratory evaluation revealed leukopenia, thrombocytopenia, and hyponatremia. Brain magnetic resonance imaging showed no evidence of acute infarction. After LP, empiric intravenous doxycycline was initiated, and complete neurologic and systemic improvement occurred within 72 hours. After 3 days, he was discharged to home to complete a 4-week course of doxycycline.

### Patient 2

A 65-year-old immunocompetent woman was admitted with severe neck and facial pain localized to the frontal and maxillary sinuses, with suspected involvement of the bilateral V1 (ophthalmic division) and V2 (maxillary division) dermatomes. Associated signs and symptoms included headache, photophobia, blurred vision, fever (temperature 103°F), nausea, and vomiting. Symptoms began ≈2 weeks after sustaining a tick bite. Initial outpatient treatment with amoxicillin/clavulanate for presumed sinusitis was ineffective, and at admission she was febrile, tachycardic, and hypotensive, meeting sepsis criteria and requiring ICU admission. After LP and CSF collection, empiric broad-spectrum antimicrobial therapy, including doxycycline, was initiated. Clinical improvement occurred within 48 hours, and she achieved complete recovery after 2 weeks of therapy.

### Patient 3

A 74-year-old immunocompetent woman was admitted for a 1-week history of intermittent headaches, photophobia, and abnormal involuntary movements characterized by akathisia and choreiform activity. On the day of admission, fever and chills developed. Laboratory evaluation revealed mild hyponatremia with an unremarkable complete blood count. After LP, empiric therapy with doxycycline and ceftriaxone was initiated. She demonstrated marked clinical improvement after the second dose and was discharged to complete a 10-day course of doxycycline. She reported residual fatigue and cognitive slowing for ≈3 months but ultimately achieved full recovery.

### Patient 4

A 57-year-old immunosuppressed man with ulcerative colitis receiving prednisone therapy (20 mg/d) was admitted with 4 days of myalgias, arthralgias, chills, sweats, and fever, followed by onset of left parietal headache. Physical examination revealed nuchal rigidity with a negative Brudzinski sign. Laboratory studies demonstrated neutropenia, thrombocytopenia, transaminitis, and elevated C-reactive protein. After CSF collection, intravenous doxycycline was initiated, resulting in complete symptom resolution within 2 days. He was discharged home to complete a 2-week course of doxycycline therapy.

### Patient 5

A 57-year-old immunosuppressed man with primary sclerosing cholangitis and gallbladder carcinoma undergoing chemotherapy was admitted with a 9-day history of headache exacerbated by light and unresponsive to over-the-counter analgesics. Associated symptoms included nausea, vomiting, photophobia, fever, chills, and neck stiffness. Head CT demonstrated no acute abnormalities. On examination, he was febrile and tachycardic, with positive Kernig and Brudzinski signs. He met sepsis criteria and required ICU admission. After LP and CSF collection, empiric broad-spectrum antimicrobial therapy, including doxycycline, was initiated. He defervesced within 48 hours of doxycycline initiation and achieved complete recovery after a 2-week course of doxycycline therapy.

### Patient 6

A 67-year-old woman was assessed for 1 day of frontal headache and fever. Initial head CT was unremarkable, and transient symptom improvement after treatment with droperidol and diphenhydramine prompted discharge. She returned the next day with worsening headache, photophobia, neck stiffness, and recurrent fever. Laboratory evaluation demonstrated pancytopenia, hyponatremia, and elevated aminotransferases. She reported extensive outdoor exposure with multiple tick bites. LP was performed and empiric doxycycline and ceftriaxone therapy initiated. She demonstrated rapid clinical improvement within 48 hours and was discharged home to complete a 10-day course of doxycycline.

### Patient 7

A 60-year-old man had influenza-like illness develop several weeks after removing an engorged tick from his lower abdomen. Symptoms included fatigue, neck pain, headache, chills, and persistent fevers refractory to over-the-counter medications. At admission, he was febrile, confused, and tachycardic. After LP, empiric doxycycline therapy was initiated. His symptoms improved within 24 hours and resolved completely by the end of a 2-week course of doxycycline therapy.

### Patient 8

A 60-year-old woman was assessed for a 1-week history of fever, severe diffuse headache radiating to the neck, photophobia, dizziness, and confusion. At admission, she was febrile and tachycardic, with pancytopenia, meeting sepsis criteria and requiring ICU admission. CSF analysis was consistent with a traumatic LP and demonstrated marked neutrophilic pleocytosis, low protein, and glucose within reference levels. Empiric broad-spectrum antimicrobial therapy was initiated, including intravenous doxycycline (initial dose of 200 mg). She completed a 2-week course of doxycycline and had a full clinical recovery.

### Patient 9

A 70-year-old woman was admitted with a 3-week history of rigors, shaking chills, diaphoresis, nausea, malaise, myalgias, and intermittent fevers after a tick bite. She subsequently had photophobia, neck stiffness, headache, and progressive somnolence develop. CSF analysis showed clear fluid with no pleocytosis and protein and glycose levels within reference ranges. She improved within 48 hours of the start of intravenous doxycycline and achieved complete recovery by day 10.

### Patient 10

A 70-year-old man with type 2 diabetes and hypertension was assessed with acute-onset confusion and inability to engage in meaningful conversation. He reported a mild frontal headache without associated photophobia, phonophobia, nausea, vomiting, or neck stiffness. On examination he was febrile and tachycardic. Laboratory studies demonstrated thrombocytopenia and leukopenia. CSF analysis demonstrated mild lymphocytic pleocytosis with proteinorachia and glucose within reference levels. He met sepsis criteria and required ICU admission. Despite empiric broad-spectrum antimicrobial therapy, fevers persisted, and encephalopathy worsened. After initiation of intravenous doxycycline (initial dose 200 mg), he defervesced within 24 hours, and his encephalopathy resolved within 72 hours. He completed a 3-week course of doxycycline therapy and recovered.

## Discussion

Among the 10 patients described in this study, 6 had clinical features consistent with meningitis, 3 with meningoencephalitis, and 1 with encephalitis. Notable neurologic findings included akathisia and left hemiparesis in 2 patients who also exhibited meningitic symptoms. Two patients were immunocompromised, and 4 required ICU admission. All patients demonstrated clinical improvement within 24–72 hours of initiating doxycycline therapy and achieved full recovery. Total duration of doxycycline therapy ranged from 10 to 28 days; most patients received 10–14 days of doxycycline therapy.

CSF profiles were variable and differed substantially from those typically observed in bacterial meningitis or meningoencephalitis (Table 1). One patient with severe meningitic symptoms requiring ICU-level care had CSF findings within reference ranges. Another patient experienced a traumatic LP resulting in neutrophilic pleocytosis and low protein levels. Because traumatic LP more commonly leads to elevated CSF protein concentrations, the low protein observed in this case was unexpected and might reflect laboratory measurement variability without clear clinical significance. In all remaining patients, mild lymphocytic pleocytosis was observed with protein and glucose levels within reference ranges. Total CSF cell counts generally were 0–50 cells/µL.

Those findings suggest that CNS symptom severity does not correlate with the degree of CSF pleocytosis or inflammation. Neurologic manifestations of anaplasmosis therefore might be mediated by systemic inflammatory responses rather than direct CNS invasion. Although human data are lacking, a canine study of meningoencephalomyelitis with concurrent anaplasmosis failed to detect pathogen nucleic acid in brain tissue or CSF, supporting the hypothesis that direct CNS invasion is unlikely (*17*). That interpretation is further supported by the limited penetration of doxycycline across the blood–brain barrier, suggesting that clinical improvement likely results from attenuation of systemic and neuroinflammatory processes rather than direct antimicrobial activity within the CNS. Doxycycline penetrates the blood–brain barrier at ≈15%–30% of serum levels. However, CNS penetration increases when inflammation is present. Penetration might be higher in cases of *Anaplasma* meningitis (*18*), but the rapid symptomatic improvement noted after doxycycline administration suggests that the primary benefit of the drug likely stems from attenuation of systemic processes rather than direct CNS activity. 

The hyperinflammatory potential of anaplasmosis is further evidenced by its association with secondary hemophagocytic lymphohistiocytosis and markedly elevated serum ferritin levels during acute infection (*19*). In our study, 2 patients with sepsis who required ICU admission and in whom anaplasmosis was suspected at admission received an initial 200-mg intravenous dose of doxycycline to increase CNS concentrations because of its limited penetration across the blood–brain barrier. However, that practice is not recommended by current guidelines (*20*), and whether it provides clinical benefit remains unclear. Patient 1 initially had stroke-like symptoms, including hemiparesis and urinary retention, in addition to symptoms of meningitis. Although neuroimaging in this patient was unrevealing and symptoms resolved with doxycycline therapy, anaplasmosis-associated stroke has been reported in other cases and is thought to result from endothelial injury (*8*,*15*,*21*,*22*).

As observed previously, our findings suggest that CNS manifestations of anaplasmosis are common but seldom reported (*7*). Although encephalitis has been described as a relatively frequent CNS manifestation (*23*), many reported cases lacked CSF evaluation, raising the possibility of encephalopathy rather than true encephalitis. As of April 2025, only 1 prior case from our institution had documented encephalitis supported by CSF findings with rapid response to doxycycline therapy (*13*). Further studies are needed to elucidate the pathophysiologic mechanisms underlying CNS involvement in anaplasmosis.

Diagnosis of anaplasmosis relies on clinical evaluation within an appropriate epidemiologic context. Most patients exhibit fever, pancytopenia (particularly thrombocytopenia), elevated transaminases, and gastrointestinal symptoms (*5*). In endemic regions, recognition of that clinical constellation often prompts empiric doxycycline therapy, resulting in rapid symptom resolution. In all our cohort patients, CNS symptoms improved within 24–72 hours of initiating doxycycline. We hypothesize that many patients improve before LP can be performed, which might explain the relatively low frequency of CSF evaluation in anaplasmosis.

Although bacterial culture remains the most sensitive diagnostic modality for anaplasmosis (*24*), routine clinical use is impractical because specialized cell culture systems are needed and the incubation period can be up to 4 weeks (*24*). Consequently, blood-based molecular diagnostic tests commonly are used (*25*). Serologic testing is frequently negative early in the course of disease and cannot reliably exclude acute infection (*24*,*25*). We identified only 1 reported case in which *Anaplasma* spp. nucleic acid was detected in CSF (*26*).

Approximately 30% of patients with anaplasmosis require hospitalization with encephalopathy identified as a risk factor (*1*). In our cohort, 4 patients with CNS symptoms required ICU admission; however, all were clinically stable for transfer to a general medical ward within 48 hours, and only 1 required vasopressor support. ICU admission remains uncommon in anaplasmosis, as demonstrated in a multicenter study in France (*27*). The higher ICU admission rate in our cohort might reflect recognition that anaplasmosis in Europe is generally a milder disease than in the United States (*28*). Genetic differences between *Anaplasma* spp. from North America and Europe might partially explain those clinical variations (*28*). Differences in strain pathogenicity, reporting practices, and underdiagnosis also might contribute to the observed disparity (*28*).

Our literature search identified 9 reported cases of anaplasmosis with CNS manifestations in which patients underwent LP and CSF examination (Table 2). Four of those patients had clinical signs and symptoms of encephalitis, 2 of meningoencephalitis, and 1 of meningitis. The other 2 patients who underwent LP had headache and CSF findings within reference ranges; they ultimately had trigeminal neuralgia and stroke diagnosed in addition to anaplasmosis. One patient with encephalitis also had concomitant ischemic stroke. Other rare neurologic manifestations, including opsoclonus-myoclonus-ataxia syndrome and hypometric saccades, have been described (*12*). Most (7/9) of the patients had CSF protein and glucose levels within reference ranges, and all 9 reported case-patients demonstrated lymphocytic pleocytosis, except for 1 patient with meningoencephalitis who had an equal percentage of neutrophils and lymphocytes in the CSF (Table 2). Although not routinely performed, metagenomic next-generation sequencing can detect *Anaplasma* spp. bacteria in CSF in select cases of meningitis or encephalitis (*26*,*29*).

**Table 2:** Characteristics of patients with anaplasmosis from published case-reports used to investigate cerebrospinal fluid findings among patients with anaplasmosis and central nervous system involvement, Minnesota and Wisconsin, USA*

Reference no.	Age, y/sex	Immune status	Neurologic presentation	ICU admission	Cerebrospinal fluid†			
					Leukocyte count	Leukocyte type (%)	Protein, mg/dL	Glucose, mg/dL
(*8*)	65/F	IS	Stroke, encephalitis	Y	**7**	Lymphocyte (78)	28	**136**
(*9*)	70/F	IC	Syncope, meningoencephalitis	NR	**10**	Lymphocyte (50); neutrophils (50)	34	57.7
(*10*)	62/M	IC	Encephalitis	Y	**10**	Lymphocyte (50)	Within reference range	Within reference range
(*11*)	80/F	IC	Trigeminal neuralgia, fever, headache	N	0	NA	20	65
(*12*)	62/M	IC	OMA, saccades, encephalitis	NR	**160**	Lymphocyte (NR)	**243**	NR
(*13*)	33/M	IC	Meningitis	NR	**4**	Lymphocyte (85)	**52**	64
(*14*)	64/F	NR	Meningoencephalitis	NR	**6**	Lymphocyte (92)	38	60
(*15*)	70/F	NR	Encephalopathy, lacunar infarct	NR	0	NA	34.7	**130.2**
(*16*)	41/M	NR	Encephalitis, seizure	NR	**4**	NR	36	71

*Bold font indicates values above reference ranges. IC, immunocompetent; ICU, intensive care unit; IS, immunosuppressed; NA, not applicable; NR, not reported; OMA, opsoclonus-myoclonus-ataxia. †Protein and glucose values were categorized as high according to reference ranges provided in the respective reports, when available. In adults, protein levels should be <50 mg/dL and glucose ranges from 36–101 mg/dL (2.0–5.6 mmol/L).

In conclusion, patients with anaplasmosis who develop meningitis, encephalitis, or meningoencephalitis typically demonstrate minimal CSF abnormalities, characterized by absent or mild pleocytosis with protein and glucose levels within reference ranges. LP and CSF analysis might be of limited use for managing anaplasmosis-associated CNS symptoms and might be more valuable for excluding alternative infectious etiologies that can also cause neurologic symptoms after a tick bite, particularly neuroborreliosis, ehrlichiosis, and Powassan virus infection.

Appendix Additional information on cerebrospinal fluid findings among patients with anaplasmosis and central nervous system involvement, Minnesota and Wisconsin, USA.

## References

[1] Katragadda S, Yetmar ZA, Chesdachai S, Fida M, Pritt BS, Challener DW, et al. Trends in anaplasmosis over the past decade: a review of clinical features, laboratory data, and outcomes. Clin Infect Dis. 2026;82:539–47. 10.1093/cid/ciaf171 40176262 PMC13169164

[2] Biggs HM, Behravesh CB, Bradley KK, et al. Diagnosis and management of tickborne rickettsial diseases: Rocky Mountain spotted fever and other spotted fever group rickettsioses, ehrlichioses, and anaplasmosis—United States. MMWR Recomm Rep. 2016;65:1–44. 10.15585/mmwr.rr6502a1 27172113

[3] Ingram D, Joseph B, Hawkins S, Spain J. Anaplasmosis in Pennsylvania: clinical features, diagnosis, and outcomes of patients diagnosed with *Anaplasma phagocytophilum* infection at Hershey Medical Center from 2008 to 2021. Open Forum Infect Dis. 2023;10:ofad193. 10.1093/ofid/ofad193 37125231 PMC10135425

[4] MacQueen D, Centellas F. Human granulocytic anaplasmosis. Infect Dis Clin North Am. 2022;36:639–54. 10.1016/j.idc.2022.02.008 36116840

[5] Dumic I, Jevtic D, Veselinovic M, Nordstrom CW, Jovanovic M, Mogulla V, et al. Human granulocytic anaplasmosis—a systematic review of published cases. Microorganisms. 2022;10:1433. 10.3390/microorganisms10071433 35889152 PMC9318722

[6] Schudel S, Gygax L, Kositz C, Kuenzli E, Neumayr A. Human granulocytotropic anaplasmosis—a systematic review and analysis of the literature. PLoS Negl Trop Dis. 2024;18:e0012313. 10.1371/journal.pntd.0012313 39102427 PMC11326711

[7] Moniuszko-Malinowska A, Dunaj J, Andersson MO, Chmielewski T, Czupryna P, Groth M, et al. Anaplasmosis in Poland—analysis of 120 patients. Ticks Tick Borne Dis. 2021;12:101763. 10.1016/j.ttbdis.2021.101763 34161867

[8] Guru S, Mahar M, Guru N, Parent L. Neurologic manifestation of anaplasmosis: a case report. Cureus. 2025;17:e77877. 10.7759/cureus.77877 39991338 PMC11846626

[9] Sohani Z, Zhao N, Weiss K, Knecht H. Anaplasmosis encephalitis and infection of non-myeloid bone marrow precursors. BMJ Case Rep. 2023;16:e254603. 10.1136/bcr-2023-254603 38035680 PMC10689420

[10] Cosiquien RJS, Stojiljkovic N, Nordstrom CW, Amadi E, Lutwick L, Dumic I. *Anaplasma phagocytophilum* encephalitis: a case report and literature review of neurologic manifestations of anaplasmosis. Infect Dis Rep. 2023;15:354–9. 10.3390/idr15040035 37489389 PMC10366838

[11] LeDonne MJ, Ahmed SA, Keeney SM, Nadworny H. Trigeminal neuralgia as the principal manifestation of anaplasmosis: a case report. Cureus. 2022;14:e21668. 10.7759/cureus.21668 35237471 PMC8882039

[12] Merati M, Rucker JC, McKeon A, Frucht SJ, Hu J, Balcer LJ, et al. A case of opsoclonus-myoclonus-ataxia with neuronal intermediate filament IgG detected in cerebrospinal fluid. J Neuroophthalmol. 2022;42:278–81. 10.1097/WNO.0000000000001599 35594157 PMC9620397

[13] Khera KD, Southerland DM, Miller NE, Garrison GM. A case of anaplasmosis during a warm Minnesota fall. J Prim Care Community Health. 2021;12:21501327211005895. 10.1177/21501327211005895 33764206 PMC8772355

[14] Mullholand JB, Tolman N, De Obaldia A, Hennrikus E. Central nervous system involvement of anaplasmosi**s.** BMJ Case Rep. 2021;14:e243665. 10.1136/bcr-2021-243665 34880034 PMC8655545

[15] Kim SW, Kim CM, Kim DM, Yun NR. Manifestation of anaplasmosis as cerebral infarction: a case report. BMC Infect Dis. 2018;18:409. 10.1186/s12879-018-3321-4 30119642 PMC6098650

[16] Young NP, Klein CJ. Encephalopathy with seizures having PCR‐positive *Anaplasma phagocytophilum* and *Ehrlichia chaffeensis.* Eur J Neurol. 2007;14:e3–4. 10.1111/j.1468-1331.2006.01582.x 17250712

[17] Barber RM, Li Q, Diniz PP, Porter BF, Breitschwerdt EB, Claiborne MK, et al. Evaluation of brain tissue or cerebrospinal fluid with broadly reactive polymerase chain reaction for *Ehrlichia, Anaplasma*, spotted fever group *Rickettsia, Bartonella*, and *Borrelia* species in canine neurological diseases (109 cases). J Vet Intern Med. 2010;24:372–8. 10.1111/j.1939-1676.2009.0466.x 20102497

[18] Nau R, Sörgel F, Eiffert H. Penetration of drugs through the blood-cerebrospinal fluid/blood-brain barrier for treatment of central nervous system infections. Clin Microbiol Rev. 2010;23:858–83. 10.1128/CMR.00007-10 20930076 PMC2952976

[19] Jevtic D, da Silva MD, Haylock AB, Nordstrom CW, Oluic S, Pantic N, et al. Hemophagocytic lymphohistiocytosis (HLH) in patients with tick-borne illness: a scoping review of 98 cases. Infect Dis Rep. 2024;16:154–69. 10.3390/idr16020012 38525759 PMC10961790

[20] Wormser GP, Dattwyler RJ, Shapiro ED, Halperin JJ, Steere AC, Klempner MS, et al. The clinical assessment, treatment, and prevention of Lyme disease, human granulocytic anaplasmosis, and babesiosis: clinical practice guidelines by the Infectious Diseases Society of America. Clin Infect Dis. 2006;43:1089–134. 10.1086/508667 17029130

[21] García-Baena C, Cárdenas MF, Ramón JF. Cerebral haemorrhage as a clinical manifestation of human ehrlichiosis. BMJ Case Rep. 2017;2017:bcr2016219054. 10.1136/bcr-2016-219054 28751428 PMC5612296

[22] Eldaour Y, Hariri R, Yassin M. Severe anaplasmosis presenting as possible CVA: case report and 3-year *Anaplasma* infection diagnosis data is based on PCR testing and serology. IDCases. 2021;24:e01073. 10.1016/j.idcr.2021.e01073 33850717 PMC8022154

[23] Kositz C, Gygax L, Schudel S, Kuenzli E, Neumayr A. Comparison of the epidemiological and clinical fingerprints of human granulocytotropic anaplasmosis and human monocytotropic ehrlichiosis in the United States. PLoS One. 2025;20:e0334957. 10.1371/journal.pone.0334957 41218048 PMC12604789

[24] Aguero-Rosenfeld ME, Zentmaier L, Liveris D, Visintainer P, Schwartz I, Dumler JS, et al. Culture and other direct detection methods to diagnose human granulocytic anaplasmosis. Am J Clin Pathol. 2025;163:313–9. 10.1093/ajcp/aqae126 39305492 PMC11821265

[25] Wormser GP, Zentmaier L, Liveris D, Schwartz I, Schneider L, Aguero-Rosenfeld ME. Antibodies to *Anaplasma phagocytophilum* in patients with human granulocytic anaplasmosis confirmed by both polymerase chain reaction and culture. Am J Med. 2025;138:669–72. 10.1016/j.amjmed.2024.11.025 39631641 PMC11925685

[26] Lee FS, Chu FK, Tackley M, Wu AD, Atri A, Wessels MR. Human granulocytic ehrlichiosis presenting as facial diplegia in a 42-year-old woman. Clin Infect Dis. 2000;31:1288–91. 10.1086/317466 11073767

[27] Gerber V, Lemmet T, Bonijoly T, Hoellinger B, Pachart A, Woerly A, et al. Retrospective multicenter study of human granulocytic anaplasmosis, France, 2012–2024. Emerg Infect Dis. 2025;31:2225–32. 10.3201/eid3112.250946 41490466 PMC12782265

[28] Matei IA, Estrada-Peña A, Cutler SJ, Vayssier-Taussat M, Varela-Castro L, Potkonjak A, et al. A review on the eco-epidemiology and clinical management of human granulocytic anaplasmosis and its agent in Europe. Parasit Vectors. 2019;12:599. 10.1186/s13071-019-3852-6 31864403 PMC6925858

[29] Piantadosi A, Mukerji SS, Ye S, Leone MJ, Freimark LM, Park D, et al. Enhanced virus detection and metagenomic sequencing in patients with meningitis and encephalitis. MBio. 2021;12:e0114321. 10.1128/mBio.01143-21 34465023 PMC8406231

