# Supplementary material for: Cerebrospinal Fluid Findings among Patients with Anaplasmosis and Central Nervous Involvement, Minnesota and Wisconsin, USA
Source: Emerg Infect Dis. 2026 Jun;32(6):844–50. doi: 10.3201/eid3206.260240 (PMC13245208; doi:10.3201/eid3206.260240)
Supplement: Appendix — Additional information on cerebrospinal fluid findings among patients with anaplasmosis and central nervous system involvement, Minnesota and Wisconsin, USA. [file 26-0240-Techapp-s1.pdf]

Article DOI: https://doi.org/10.3201/eid3206.260240

EID cannot ensure accessibility for supplementary materials supplied by authors. Readers who have difficulty accessing supplementary content should contact the authors for assistance.

Cerebrospinal Fluid Findings among Patients with Anaplasmosis and Central Nervous Involvement, Minnesota and Wisconsin, USA

Appendix

Appendix Table. Cerebrospinal fluid (CSF) meningitis/encephalitis panel results for study patients*

Pathogen	Test	Result
Herpes simplex virus type 1 and 2	PCR	Negative
Varicella zoster virus	PCR	Negative
Cytomegalovirus	PCR	Negative
Human parechovirus	PCR	Negative
Human herpesvirus 6	PCR	Negative
Epstein–Barr virus	PCR	Negative
Enterovirus 71	PCR	Negative
Adenovirus	PCR	Negative
Lymphocytic choriomeningitis virus	IgM and IgG	Negative
West Nile virus	IgM and IgG	Negative
Jamestown Canyon virus	IgM	Negative
St. Louis encephalitis virus	IgM and IgG	Negative
California (La Crosse) encephalitis virus	IgM and IgG	Negative
Western equine encephalitis virus	IgM and IgG	Negative
Eastern equine encephalitis virus	IgM and IgG	Negative
Powassan virus	IgM and IgG	Negative
Escherichia coli K1	PCR	Negative
Neisseria meningitidis	PCR	Negative
Borrelia burgdorferi	PCR, IgM, and IgG	Negative
Leptospira spp.	IgM and IgG	Negative
Listeria monocytogenes	PCR	Negative
Streptococcus pneumoniae	PCR	Negative
Streptococcus agalactiae	PCR	Negative
Hemophilus influenzae	PCR	Negative
Cryptococcus neoformans/gattii	PCR	Negative

*All testing was performed on cerebrospinal fluid samples as part of the routine meningitis/encephalitis diagnostic panel. Results for all listed pathogens were negative for all 10 patients included in the study.
